# Ferroptosis in Neuropsychiatric and Neurodegenerative Disorders: Shared Mechanisms and Disease-Specific Signatures

**DOI:** 10.3390/ph19040629

**Published:** 2026-04-16

**Authors:** Mingxin Liu, Chen Zeng, Zizhen Si

**Affiliations:** Department of Medicine, Ningbo University, Ningbo 315211, China

**Keywords:** ferroptosis, lipid peroxidation, oxidative stress, mental disorders, neurodegeneration

## Abstract

Neuropsychiatric and neurodegenerative disorders impose a substantial global health burden, yet progress in mechanism-based therapy remains limited by clinical heterogeneity and an incomplete understanding of disease biology. Emerging evidence implicates ferroptosis—an iron-dependent form of lipid peroxidation-driven cell death—as a shared pathogenic process across primary psychiatric disorders and neurodegenerative diseases with prominent neuropsychiatric features. In this review, we synthesize evidence from major depressive disorder, schizophrenia, substance use disorders, Alzheimer’s disease (AD), and Parkinson’s disease (PD), highlighting ferroptosis as a common mechanism linking iron dyshomeostasis to neuronal dysfunction. Mechanistically, ferroptosis is organized around three interconnected modules: amino acid metabolism, lipid peroxidation, and iron handling. These pathways converge on mitochondrial dysfunction, oxidative damage, and neuroinflammatory amplification. We further propose that each disorder displays a distinct ferroptosis signature, including dopamine quinone-mediated GPX4 loss in PD, AICD-dependent transcriptional reprogramming in AD, and inflammatory–glutamatergic lowering of the ferroptotic threshold in depression and schizophrenia. Together, these insights position ferroptosis as a candidate framework for biomarker development, patient stratification, and mechanism-informed therapeutic intervention across neuropsychiatric disease.

## 1. Introduction

Ferroptosis was formally described in 2012 after the observation that small molecules such as erastin selectively induced death in RAS-mutant tumour cells. Since then, ferroptosis has been recognized as a broader biological process with important implications far beyond cancer, including in disorders of the central nervous system [1]. Ferroptosis is a regulated form of cell death driven by iron-dependent phospholipid peroxidation. It is shaped by three interconnected processes: iron accumulation, lipid peroxidation, and failure of antioxidant defence systems [2]. Ferroptosis is closely linked to mitochondrial dysfunction, oxidative stress, autophagy and crosstalk with apoptotic signalling pathways [3]. Iron overload can lead to serious consequences such as hypoxia, tissue damage, and multi-organ failure. Although iron is an important micronutrient in the brain, participating in the catalysis of many enzymes, regulating the synthesis and metabolism of neurotransmitters, and influencing neuronal activity and plasticity, excessive amounts can be harmful [2].

Mental disorders are characterized by persistent or recurrent disturbances in emotion, cognition and behaviour that impair daily functioning and quality of life [4]. Mental disorders can alter diet, nutrient intake and medication exposure, all of which may influence systemic and cerebral iron homeostasis. The prevalence and burden of mental disorders continue to increase globally, making them a significant global issue.

**Figure 1:**
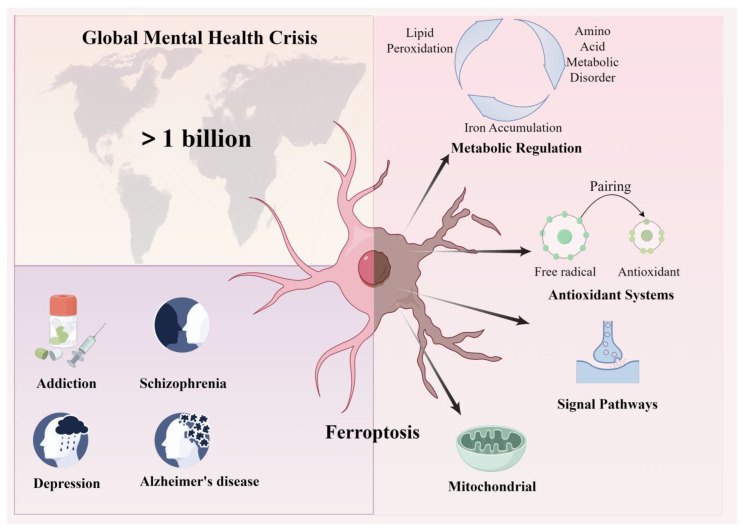
Core Ferroptosis Pathways and Their Relevance to Neuropsychiatric Disorders.

Iron deficiency can impact the oxygenation and functioning of the brain, potentially leading to various mental disorders such as depression, anxiety, and schizophrenia [5]. Mental disorders may be accompanied by iron deficiency and malabsorption when affecting an individual’s diet and nutritional intake. In addition, iron metabolism may be perturbed by disease-associated oxidative stress or by pharmacological interventions, thereby altering susceptibility to ferroptosis [5] (Figure 1). The relationship between iron imbalance and mental disorders is likely bidirectional, but the available evidence remains fragmented and has not yet converged on a unified mechanistic framework. This review examines how ferroptosis intersects with psychiatric and neurodegenerative disorders, highlights shared and disease-specific mechanisms, and discusses the therapeutic opportunities and unresolved challenges in this rapidly evolving field.

Ferroptosis is driven by three interconnected processes: iron accumulation, impaired cystine–glutathione–GPX4 antioxidant defence, and phospholipid peroxidation. In the central nervous system, disruption of these pathways can compromise mitochondrial function, amplify oxidative stress and neuroinflammation, and thereby contribute to neuronal dysfunction across addiction, depression, schizophrenia and neurodegenerative disease. Arrows indicate proposed bidirectional links between disturbed brain iron homeostasis and neuropsychiatric pathology.

## 2. Mechanisms of Ferroptosis

Ferroptosis is a distinct form of regulated cell death characterized by iron-dependent peroxidation of membrane phospholipids. Morphologically, biochemically and genetically, it differs from apoptosis, necroptosis, pyroptosis and autophagy-dependent cell death [6]. Disturbed intracellular amino acid metabolism, lipid peroxidation, and iron accumulation are the three central biochemical events that lead to ferroptosis [7]. The regulation of iron metabolism and redox balance involves organelles such as mitochondria and lysosomes [8].

### 2.1. Metabolic Regulation

#### 2.1.1. Amino Acid Metabolism and the System Xc^−^–GSH–GPX4 Axis

**Figure 2:**
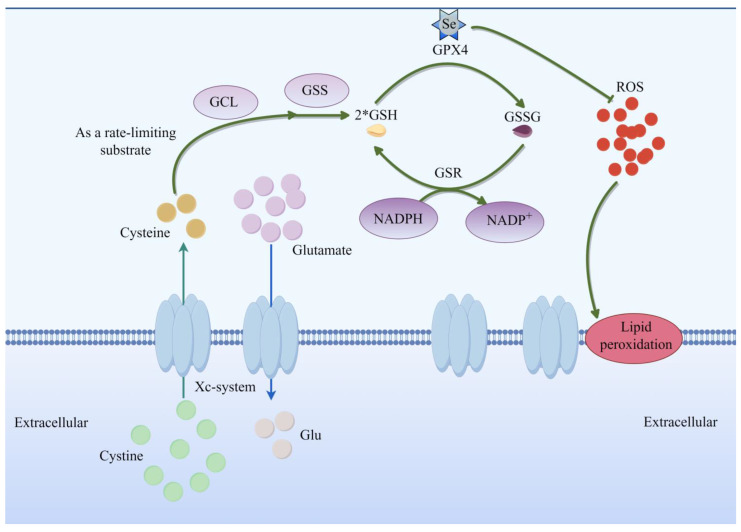
Mechanisms of Amino Acid Metabolic Disorder.

System Xc^−^, a cystine/glutamate antiporter, mediates the 1:1 exchange of extracellular cystine and intracellular glutamate [9,10]. This process is critical for maintaining intracellular cystine levels, which are subsequently reduced to cysteine (Cys) through NADPH-dependent or thioredoxin-mediated pathways. Cysteine then serves as a rate-limiting substrate for the synthesis of glutathione (GSH) [11]. Glutathione peroxidase 4 (GPX4), a selenoprotein, utilizes GSH as a cofactor to reduce phospholipid hydroperoxides (PL-PUFAs-OOH) to their corresponding alcohols (PL-PUFAs-OH), thereby preventing lipid peroxidation. However, when GSH is depleted or GPX4 is inactivated, phospholipid hydroperoxides accumulate. In the presence of Fe^2+^, these oxidized lipids promote radical propagation and membrane damage, ultimately triggering ferroptosis [12]. This generates hydroxyl radicals (-OH), which propagate lipid peroxidation chain reactions, leading to membrane bilayer destabilization and iron-dependent cell death—the hallmark of ferroptosis. Consistent with this model, erastin blocks system Xc^−^ activity, depletes intracellular cysteine and glutathione, and thereby induces ferroptosis [13]. In subsequent anticancer drug research, sulfasalazine (SAS) was found to inhibit the system Xc^−^ subunit SLC7A11, thereby reducing cystine uptake, depleting intracellular cysteine and glutathione, and promoting ferroptosis [14]. Glutamate can also reduce intracellular Cys levels by inhibiting the transport of the system Xc^−^, resulting in blocked GSH synthesis and the induction of ferroptosis [13] (Figure 2).

This figure illustrates in detail the central role of amino acid metabolic disorders, particularly the system Xc^−^-glutathione (GSH)–glutathione peroxidase 4 (GPX4) signaling axis, in the regulation of ferroptosis. System Xc^−^ is a cystine/glutamate antiporter located on the cell membrane that transports extracellular cystine into the cell while simultaneously exporting intracellular glutamate at a 1:1 ratio. Once inside the cell, cystine is reduced to cysteine through thioredoxin- or NADPH-dependent pathways. Cysteine then serves as the rate-limiting substrate for the synthesis of GSH, catalyzed sequentially by glutamate-cysteine ligase (GCL) and glutathione synthetase (GSS). GSH functions as an essential cofactor for GPX4, a selenium (Se)-containing enzyme that reduces toxic lipid peroxides to non-toxic lipid alcohols, thereby interrupting the lipid peroxidation chain reaction and suppressing ferroptosis. When system Xc^−^ is blocked by inhibitors (such as Erastin or sulfasalazine, SAS) or by high concentrations of glutamate, intracellular cystine uptake is reduced, leading to GSH depletion, decreased GPX4 activity, and accumulation of lipid peroxides. These accumulated lipid peroxides, under the mediation of ferrous iron (Fe^2+^)-dependent Fenton reactions, generate substantial reactive oxygen species (ROS), ultimately triggering ferroptosis. Additionally, the figure depicts the GSH regeneration cycle: oxidized glutathione (GSSG) is reduced back to GSH by glutathione reductase (GSR) using NADPH as the reducing equivalent donor—a cycle critical for maintaining cellular antioxidant capacity.

#### 2.1.2. Lipid Peroxidation

**Figure 3:**
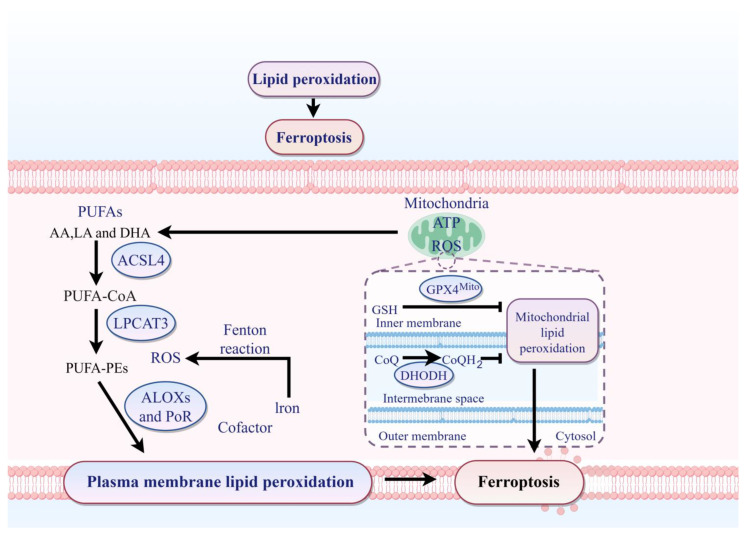
Mechanisms of Lipid Peroxidation.

Ferroptosis execution hinges on the iron-catalyzed peroxidation of polyunsaturated fatty acid (PUFA)-containing phospholipids (PUFA-PLs), a process precisely regulated by three enzymatic nodes: acyl-CoA synthetase long-chain family member 4 (ACSL4), lysophosphatidylcholine acyltransferase 3 (LPCAT3), and lipoxygenases (LOXs) [15]. ACSL4 governs the initiation of this cascade by selectively activating ω-6 polyunsaturated fatty acids (e.g., arachidonic acid [AA] and adrenic acid [AdA]) into acyl-CoA esters [16,17]. This substrate specificity enriches cellular membranes in oxidation-prone PUFA-CoA species. LPCAT3 then incorporates these activated fatty acids into membrane phospholipids through esterification, generating AA-phosphatidylethanolamines (AA-PEs) and AdA-phosphatidylethanolamines (AdA-PEs) [12]. By anchoring PUFA to phospholipids, LPCAT3 spatially confines peroxidation targets to lipid bilayers, amplifying oxidative damage efficiency. LOXs act as the terminal oxidoreductases, directly converting AA-PEs and AdA-PEs into lipid hydroperoxides (PL-OOH) [17,18]. The accumulation of PL-OOH initiates self-propagating lipid radical chain reactions, culminating in catastrophic membrane rupture—the biochemical hallmark of ferroptosis. Several small molecules suppress ferroptosis by limiting lipid peroxide accumulation. For example, ferrostatin-1 inhibits erastin- or RSL3-induced ferroptosis and can also rescue p53-driven ferroptotic phenotypes by blocking lipid peroxidation [19,20,21] (Figure 3).

This figure depicts the core enzymatic cascade driving lipid peroxidation and the subcellular compartmentalization of ferroptosis regulation. The peroxidation cascade is initiated by **acyl-CoA synthetase long-chain family member 4 (ACSL4)**, which esterifies polyunsaturated fatty acids (PUFAs)—including arachidonic acid (AA), linoleic acid (LA), and docosahexaenoic acid (DHA)—into PUFA-CoA. **Lysophosphatidylcholine acyltransferase 3 (LPCAT3)** subsequently incorporates these PUFA-CoA into membrane phospholipids to generate PUFA-phosphatidylethanolamines (PUFA-PEs). The terminal oxidation of PUFA-PEs is catalyzed by **lipoxygenases (ALOXs)** and **cytochrome P450 oxidoreductase (PoR)**, producing lipid hydroperoxides that destabilize membrane integrity. Mitochondria function as critical regulatory hubs through multiple parallel systems: **GPX4Mito** (mitochondrial GPX4) utilizes GSH to detoxify lipid peroxides; **dihydroorotate dehydrogenase (DHODH)** operates at the inner membrane to limit mitochondrial lipid peroxidation; and the **coenzyme Q (CoQ)/ubiquinol (CoQH_2_)** system serves as an endogenous lipophilic antioxidant regenerated by mitochondrial enzymes. In the intermembrane space and cytosol, ferrous iron (Fe^2+^) catalyzes **Fenton reactions**, generating hydroxyl radicals that initiate non-enzymatic lipid peroxidation. Plasma membrane lipid peroxidation—whether initiated enzymatically by ALOXs/PoR or non-enzymatically by Fenton chemistry—leads to membrane rupture and ferroptotic cell death.

#### 2.1.3. Iron Accumulation

Disrupted iron trafficking is a core determinant of ferroptosis. At the cellular level, ferroptotic vulnerability is shaped by iron uptake, storage, mobilization and export, all of which regulate expansion of the labile iron pool (LIP).

Transferrin-Bound Iron Uptake (TBI Pathway)

Circulating Fe^3+^ binds to transferrin (Tf) to form a complex, which is recognized by transferrin receptor 1 (TfR1) on the cell membrane and internalized via receptor-mediated endocytosis. Notably, TfR1 serves as the primary iron uptake pathway for most cells, whereas TfR2, predominantly expressed in hepatocytes and erythroid precursor cells, is implicated in iron homeostasis regulation rather than direct iron uptake [22,23]. Within the acidic endosomal environment, Fe^3+^ dissociates from transferrin and is reduced to Fe^2+^ by metalloreductases such as six-transmembrane epithelial antigen of prostate 3 (STEAP3) or duodenal cytochrome b (DCYTB). The reduced Fe^2+^ is then transported into the cytoplasm via divalent metal transporter 1 (DMT1) [22,24].

Non-Transferrin-Bound iron uptake (NTBI Pathway)

When plasma iron concentrations exceed the binding capacity of transferrin, free iron (NTBI) enters cells directly through membrane transporters SLC39A14 and SLC39A8. Intracellular NTBI is further transported into the cytoplasm via DMT1 or ZIP14 from endosomal membranes. This pathway is particularly significant in iron overload disorders, such as hereditary hemochromatosis [23,25,26].

Regulation of Iron Export and Storage

Ferroportin (FPN/SLC40A1), the sole known iron exporter, maintains intracellular iron homeostasis by effluxing Fe^2+^ to the extracellular space, where it is oxidized to Fe^3+^ by ceruloplasmin [23]. Additionally, the iron storage protein ferritin sequesters free Fe^2+^ to prevent oxidative damage. Ferritin degradation is regulated by nuclear receptor coactivator 4 (NCOA4)-mediated ferritinophagy. Overactivation of NCOA4 accelerates ferritin degradation, resulting in excessive Fe^2+^ release into the LIP [7].

Iron Accumulation-Driven Ferroptosis

Iron dyshomeostasis drives oxidative stress through coordinated disruption of iron buffering, trafficking and storage systems. Under physiological conditions, iron is safely transported in circulation as TBI, which restricts redox activity and ensures regulated cellular uptake via transferrin receptor-dependent pathways [27,28]. When transferrin capacity is exceeded or impaired, NTBI emerges as a heterogeneous and redox-active iron pool that bypasses canonical regulatory checkpoints and enters cells through alternative transporters such as ZIP14 and ZIP8, thereby expanding the labile iron pool (LIP) [27,28,29]. This unrestrained iron influx promotes Fe^2+^-dependent Fenton chemistry, resulting in excessive ROS generation, lipid peroxidation and mitochondrial dysfunction [28,29,30]. Consistently, experimental models demonstrate that transferrin deficiency elevates NTBI levels and enhances ferroptosis-associated tissue injury, whereas inhibition of NTBI uptake mitigates oxidative damage [27,29].

At the intracellular level, ferritin functions as the principal iron storage complex, sequestering excess iron in a mineralized and redox-inert form to prevent its participation in ROS-generating reactions [31,32]. Disruption of ferritin integrity or enhanced ferritin turnover leads to expansion of the LIP, increasing the availability of redox-active Fe^2+^ and amplifying oxidative stress [31,33]. Central to this process is NCOA4, which acts as a selective cargo receptor for ferritinophagy and mediates lysosomal degradation of ferritin, thereby mobilizing stored iron into the cytosol [6]. NCOA4-dependent ferritin turnover directly increases intracellular free iron levels, promoting Fenton-driven ROS production and lipid peroxidation [33]. Under iron-replete conditions, NCOA4 is ubiquitinated and degraded, limiting ferritin turnover and preventing excessive iron mobilization; conversely, NCOA4 upregulation or ferritin depletion drives excessive iron release, contributing to mitochondrial dysfunction, ROS accumulation and increased ferroptotic susceptibility [32,33].

**Figure 4:**
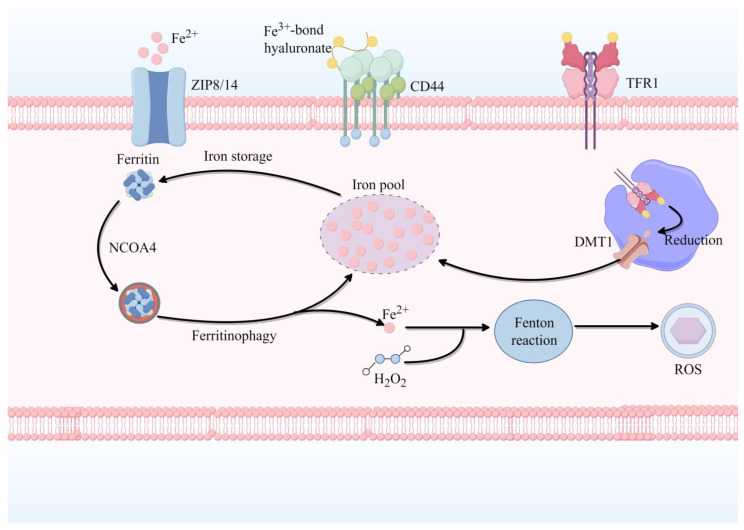
Iron Accumulation.

Dysregulation of iron transport and storage systems further exacerbates this process. Upregulation of iron importers such as transferrin receptor 1 (TfR1) and divalent metal transporter 1 (DMT1), together with impaired iron export via ferroportin (FPN) or ferritin dysfunction, results in pathological accumulation of Fe^2+^ in the LIP [34,35]. This redox-active iron catalyzes ROS production via the Fenton reaction, initiating lipid peroxidation and ultimately driving ferroptotic cell death [36]. Pharmacological interventions underscore the central role of iron flux in this cascade: iron chelators such as deferoxamine (DFO) reduce LIP and suppress ROS generation and ferroptosis, whereas activation of NCOA4-mediated ferritinophagy enhances iron release and exacerbates oxidative injury [33,37]. Collectively, these findings support a unified model in which loss of extracellular iron buffering (TBI → NTBI), intracellular iron sequestration (ferritin), and controlled iron recycling (NCOA4) converges to convert iron from a tightly regulated cofactor into a catalytically active driver of oxidative stress and ferroptotic cell death (Figure 4).

This figure illustrates the cellular iron trafficking pathways that converge on ferrous iron (Fe^2+^) accumulation in the labile iron pool (LIP), ultimately driving ferroptosis through Fenton chemistry. Iron uptake occurs through two primary routes: the **transferrin-bound iron (TBI) pathway** and **non-transferrin-bound iron (NTBI) pathway**. In the TBI pathway, extracellular Fe^3+^ bound to transferrin is recognized by **transferrin receptor 1 (TFR1)** on the plasma membrane and internalized via endocytosis. Within endosomes, Fe^3+^ is reduced to Fe^2+^ and transported into the cytosol by **divalent metal transporter 1 (DMT1)**. In the NTBI pathway, free iron enters cells directly through **ZIP8/14** transporters, with **CD44**-hyaluronate interactions potentially modulating this process. Intracellular iron homeostasis is maintained through storage in **ferritin**, which sequesters excess iron in a non-toxic form. However, **nuclear receptor coactivator 4 (NCOA4)** mediates **ferritinophagy**—the selective autophagic degradation of ferritin—releasing stored iron back into the LIP. The resulting elevation of cytosolic Fe^2+^ catalyzes the **Fenton reaction**, converting hydrogen peroxide (H_2_O_2_) into highly reactive hydroxyl radicals (•OH). These radicals initiate lipid peroxidation chain reactions, culminating in ferroptotic cell death. Dysregulation at any node—enhanced iron uptake (TFR1/DMT1 upregulation, ZIP8/14 activation), impaired storage (ferritin dysfunction), or excessive ferritinophagy (NCOA4 overactivation)—contributes to LIP expansion and ferroptosis susceptibility.

### 2.2. Antioxidant Systems

#### 2.2.1. GPX4 Pathway

Glutathione peroxidase 4 (GPX4) is a selenoprotein that uses glutathione (GSH) to reduce phospholipid hydroperoxides to the corresponding lipid alcohols, thereby suppressing ferroptosis [38,39]. Upon entering the cell, cystine (Cys2) is reduced to cysteine (Cys), which is then used for glutathione synthesis. Cys is synthesised into GSH, catalysed sequentially by glutamate-Cys ligase (GCL) and GSH synthetase (GSS) [39]. GSH serves as the essential reducing cofactor for GPX4, enabling the enzyme to convert phospholipid hydroperoxides (PL-OOH) into non-toxic phospholipid alcohols (PL-OH), thereby protecting cells from PL-OOH-induced iron toxicity [40,41]. The catalytic reaction of GPX4 follows a ping-pong mechanism in which the active site of the enzyme oscillates between the oxidised and reduced states [2]. Initially, the active site selenate (Se-H) of GPX4 is oxidised to selenate (Se-OH) by a peroxide substrate. Subsequently, the initial GSH is employed to reduce selenate, resulting in the formation of an intermolecular selenosulfur bond. The second GSH then forms a disulfide bond (-S-S-) with the initial GSH via a sulfur group (-SH), thereby generating oxidised glutathione (GSSG) and restoring GPX4 to its original state [2,41]. In the presence of reduced NADPH, which acts as an electron donor, GSSG can be reduced by glutathione reductase (GR) to regenerate GSH [42]. GPX4 inhibitors directly inhibit the activity of GPX4, leading to the accumulation of ROS and the induction of ferroptosis. RAS-selective lethal small molecule 3 (RSL3) does not affect intracellular GSH synthesis but directly inhibits the target protein GPX4, resulting in its loss of activity and the initiation of ferroptosis [43]. The small molecule FIN56 promotes the degradation of the GPX4 protein and reduces the synthesis of the lipophilic antioxidant Coenzyme Q10 (CoQ10) through the MVA pathway, thereby weakening the inhibitory effect of CoQ10 on lipid ROS generation and inducing ferroptosis. Overexpression of GPX4 in cells inhibits cell death caused by FIN56 [44]. Small molecule inhibitors that affect the GSH/GPX4 axis include β-Mercaptoethanol (β-ME), which can react with cysteine to form mixed disulfide bonds [13]. These mixed disulfide bonds are transferred into cells through the system Xc^−^, where they rapidly produce cysteine, significantly increasing the rate of GSH production and enhancing the antioxidant capacity of the GPX4 system [45].

#### 2.2.2. FSP1-CoQ10 Pathway

Coenzyme Q10 is present in various cell membranes, including mitochondria, and constitutes a second endogenous mechanism for protecting cell membranes against lipid peroxidation [46]. Lipid peroxidation in turn drives ferroptosis. The ferroptosis suppressor protein 1 (FSP1) is localised to the plasma membrane. Plasma membrane localization is required for the ferroptosis-suppressive activity of FSP1 [47,48]. As an NAD(P)H-dependent flavoprotein oxidoreductase [49], FSP1 reduces ubiquinone (CoQ10) to produce a reduced form of CoQ10 ubiquinol (CoQ10-H2), which scavenges lipid peroxidation radicals and inhibits lipid peroxidation, thereby preventing the ferroptosis response [50,51]. In certain instances, FSP1 inhibits iron-induced cell death by activating the membrane repair function of the endosomal tri-complex III (ESCRT-III), which is essential for transport, rather than its own oxidoreductase function [23,52]. Regarding FSP1 inhibitors, icFSP1, a specific drug targeting human FSP1, induces phase separation of FSP1 through N-terminal myristoylation, different amino acid residues, and inherent disorder and low complexity regions. This causes FSP1 to detach from the membrane and form droplet-like aggregates, altering its spatial location and physical conformation. Consequently, its protective effect against lipid oxidation is lost, leading to the enhanced induction of ferroptosis [53].

#### 2.2.3. DHODH–CoQ Pathway

The mitochondrial defence mechanism against ferroptosis, mediated by dihydronicotinic acid dehydrogenase (DHODH), inhibits ferroptosis by reducing CoQ on the inner mitochondrial membrane (IMM) to coenzyme QH2 (CoQH2) [54]. DHODH is localized to the inner mitochondrial membrane and opposes GPX4 inhibitor-induced ferroptosis by reducing CoQ to CoQH2. In this context, its substrate dihydroorotate acts as a ferroptosis antagonist, whereas the product orotate can enhance ferroptotic sensitivity [23].

### 2.3. Signal Pathways

#### 2.3.1. Keap1–Nrf2 Pathway

The Keap1–Nrf2 pathway is a central transcriptional defence programme against ferroptosis because it coordinately regulates antioxidant capacity, iron metabolism and lipid redox homeostasis [23,55]. It binds to the Antioxidant Response Element (ARE) in the promoter regions of numerous cytoprotective genes. Keap1 senses ROS through cysteine residues, undergoes a conformational change, and releases Nrf2 [56]. Among these, nucleophilic thiols, including Keap1 cysteine sulfhydryl groups, covalently bind to a variety of electrophilic Nrf2-inducing factors [57]. In the absence of oxidative stress, Nrf2 is modified by ubiquitination of the Keap1 (Kelch-like ECH-associated protein 1) complex and subsequently degraded in the cytoplasm. However, in response to oxidative stress, binding of p62 to Keap1 drives binding of microtubule-associated protein 1A/1B light chain 3 (LC3), which leads to degradation of Keap1 and inhibition of Keap1 complex activity [58]. This facilitates the formation of heterodimers with a small class of Maf proteins and the aggregation of Nrf2 in the nucleus [59]. Additionally, Nrf2 is involved in the regulation of β-catenin protein enhancer activity, as well as the transcriptional activation of the β-catenin cluster genes. This transcription factor protects cells from lipid peroxidation-induced ferroptosis by participating in the regulation of iron metabolism and the antioxidant system [57].

#### 2.3.2. p53-Dependent Regulation of Ferroptosis

The protein p53, officially designated as tumor protein p53, functions as a tumor suppressor. This protein regulates cellular stress responses and modulates target gene expression, ultimately driving critical cellular outcomes including cell cycle arrest, apoptosis, senescence, DNA repair, and metabolic reprogramming [60]. Notably, p53 demonstrates context-dependent dual roles in metabolic regulation, exemplified by its paradoxical functions in redox homeostasis. Under basal ROS conditions, p53 exhibits antioxidant properties that mitigate oxidative damage and promote cell survival. Conversely, when ROS exceeds critical thresholds that threaten cellular integrity, p53 amplifies oxidative stress to eliminate severely compromised cells through regulated cell death, thereby protecting neighboring healthy tissues [61].

While p53’s oncological implications have been extensively investigated, emerging evidence reveals its significant connections with ferroptosis through multiple molecular targets. A key mediator is solute carrier family 7 member 11 (SLC7A11), the functional subunit of the system Xc^−^. SLC7A11-mediated cystine uptake sustains GSH biosynthesis, which is essential for glutathione peroxidase 4 (GPX4)-dependent ferroptosis suppression [62]. Vitamin K epoxide reductase complex subunit 1 like 1 (VKORC1L1) demonstrates unique ferroptosis-inhibitory capacity through lipid peroxidation scavenging, operating independently of FSP1. Intriguingly, VKORC1L1 synergizes with other vitamin K-related pathways to enhance ferroptosis resistance [63]. Phospholipid transfer protein (PLTP) exhibits concentration-dependent dual effects: low PLTP levels promote lipid droplet formation and ferroptosis protection, while elevated levels facilitate lipid import to pro-ferroptotic thresholds. Mechanistically, PLTP’s functional dichotomy may stem from its cargo specificity—potentially transporting either ferroptosis-promoting phosphatidylethanolamine or anti-ferroptotic α-tocopherol [64].

#### 2.3.3. NMDA–NO/H_2_S Signaling in Ferroptosis Regulation

Ferroptosis—an iron-dependent form of regulated cell death driven by lipid peroxidation—has emerged as a critical contributor to neurodegeneration, particularly in the central nervous system, where high oxygen consumption, abundant iron stores and lipid-rich membranes create intrinsic vulnerability [13]. This vulnerability is directly coupled to excitatory neurotransmission through the NMDA receptor, whose overactivation is a well-established driver of neuronal death [65]. Although eNOS (NOS III) has traditionally been viewed as a Ca^2+^-dependent enzyme in endothelial cells, recent studies have conclusively demonstrated its constitutive expression in neurons, where it acts as a Ca^2+^-sensitive NO synthase that rapidly responds to glutamate-receptor-triggered Ca^2+^ influx [66,67,68]. Binding of glutamate to NMDA receptors opens the receptor channel, allowing substantial Ca^2+^ entry—the initiating event in excitotoxicity. Using rat striatal primary neuron-glia co-cultures, Strijbos et al. found that a brief (5 min) exposure to 100 μM NMDA triggered a sustained accumulation of NO metabolites over 16 h, whereas neuronal death began only after a delay of approximately 4 h; addition of the NMDAR antagonist AP5 or dizocilpine immediately after NMDA exposure completely blocked both NO accumulation and cell death, demonstrating that NMDAR-mediated Ca^2+^ influx activates Ca^2+^-dependent NOS isoforms (including eNOS) in a sustained manner, generating large amounts of NO [69]. Once produced, NO feeds back to promote further Ca^2+^ entry mainly through protein S-nitrosylation of NMDARs—under physiological conditions this modification provides negative feedback, whereas under pathological conditions (e.g., increased oxidative stress) it may enhance NMDAR activity [70]; additionally, NO can activate soluble guanylyl cyclase to produce cGMP, which in turn activates PKG and phosphorylates voltage-gated Ca^2+^ channels, indirectly augmenting Ca^2+^ entry [71]. Acting as a retrograde messenger, NO also diffuses to presynaptic terminals to promote glutamate release, establishing a self-amplifying loop that constitutes the core of the excitotoxic vicious cycle [69,71,72]. Critically, NMDA receptor stimulation triggers calcium influx that activates nNOS through the scaffold protein PSD-95, leading to NO production; NO then S-nitrosylates Dexras1, converting it to an active GTP-bound state via the adaptor protein CAPON [73]. Activated Dexras1 binds PAP7, which interacts with the divalent metal transporter DMT1, forming a ternary complex that markedly increases both non-transferrin-bound and transferrin-bound iron uptake [74]. In primary cortical neurons, this NMDA–NO–Dexras1–DMT1 cascade drives iron influx in a concentration-dependent manner—an effect blocked by the NMDA antagonist MK801 and absent in nNOS knockout mice—and under excitotoxic conditions, exposure to 300 μM NMDA elicits a pronounced increase in hydroxyl radical formation that is prevented by the cell-permeable iron chelator salicylaldehyde isonicotinoyl hydrazone (SIH), with SIH pretreatment providing near-complete protection against NMDA-induced neuronal death [74]. Thus, NO-dependent iron influx through Dexras1 and DMT1 sensitizes neurons to ferroptosis. In parallel, hydrogen sulfide (H_2_S) and its oxidized derivatives, polysulfides, act as endogenous protective signals that counteract ferroptosis through multiple converging mechanisms: they enhance system Xc^−^ activity to increase glutathione synthesis, activate Nrf2 to upregulate glutamate–cysteine ligase and heme oxygenase-1, directly scavenge lipid peroxyl radicals to terminate lipid peroxidation, and activate TRPA1 channels which regulate neurotransmitter release and sulfur metabolism in a feedback loop [75,76,77]. The interplay between NO and H_2_S signals is complex and context-dependent: they may converge on the same cysteine residues with opposing functional consequences—S-nitrosylation often being inhibitory while persulfidation is activating—and H_2_S can both enhance NMDA receptor activity under physiological conditions and dampen excitotoxicity by promoting inhibitory neurotransmitter release or activating K_ATP channels [75]. Together, these opposing pathways establish a conceptual framework in which the balance between NO-mediated iron influx and H_2_S-mediated antioxidant defense determines the threshold for ferroptotic cell death in neurons, with important implications for neurodegenerative diseases characterized by both excitotoxicity and iron accumulation [70,78].

### 2.4. Mitochondrial Metabolism and Ferroptosis

Mitochondria influence ferroptosis at three levels: substrate supply for lipid synthesis, ROS generation during oxidative phosphorylation, and compartment-specific antioxidant defence. The mitochondrial tricarboxylic acid cycle (TCA cycle) links the catabolism of carbohydrates, lipids and amino acids, providing crucial reducing equivalents for the electron transport chain (ETC). Its operational state profoundly influences the cell’s susceptibility to ferroptosis. Restricting pyruvate oxidation by inhibiting pyruvate dehydrogenase (PDH) activity suppresses glucose-dependent ferroptosis, suggesting that the carbon skeleton generated by the TCA cycle for fatty acid synthesis may indirectly promote ferroptosis [79]. Conversely, inhibition of the mitochondrial pyruvate transporter MPC1 increases the susceptibility to ferroptosis in cells dependent on glutamine metabolism [80].

Key enzymes in the tricarboxylic acid cycle act as regulatory hubs. Mitochondrial NADP^+^-dependent isocitrate dehydrogenase 2 (IDH2) generates NADPH, which maintains GSH levels via glutathione reductase (GR), thereby ensuring the function of GPX4 [81]. Downregulation of IDH2 impairs antioxidant defence and enhances susceptibility to ferroptosis. Conversely, the α-ketoglutarate dehydrogenase complex (α-KGDH) and its E3 subunit, dihydrolipoyl dehydrogenase (DLD), are also associated with ferroptosis [82].

The TCA cycle is tightly coupled with glutaminolysis. Sustained glutaminolysis is essential for driving lipid ROS accumulation under cystine deprivation [83]. Blocking glutamine metabolism effectively suppresses ferroptosis, while α-KG supplementation can bypass this inhibition [83]. The urea cycle enzyme ASS1 confers ferroptosis resistance by promoting reductive carboxylation of glutamine, diverting it from oxidative TCA metabolism [84].

**Figure 5:**
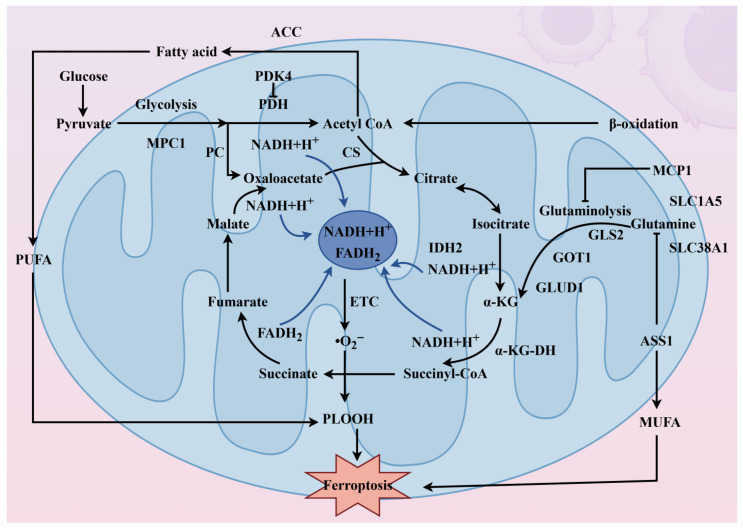
Mitochondrial Regulation of Ferroptosis.

Mitochondria perform oxidative phosphorylation (OXPHOS) via the ETC, and ETC activity and directly influences ferroptosis in an inducer-specific manner. Inhibition of ETC complexes suppresses ferroptosis induced by cystine deprivation or system Xc^−^ inhibitors, but not by direct GPX4 inhibitors [85]. AMP-activated protein kinase (AMPK) exerts dual regulatory effects on ferroptosis: it can suppress ferroptosis by limiting lipid synthesis under glucose starvation [86], or promote ferroptosis by phosphorylating Beclin 1 to inhibit system Xc^−^ [87]. AMPK also promotes pyrimidine synthesis, generating dihydroorotate (DHOA), which is converted to orotate by DHODH, linking AMPK to mitochondrial antioxidant defense [54,88] (Figure 5).

This figure depicts the central role of mitochondrial metabolism—particularly the tricarboxylic acid (TCA) cycle and its associated pathways—in modulating cellular susceptibility to ferroptosis. Mitochondria integrate multiple fuel sources that converge on the TCA cycle: glucose enters via glycolysis, with pyruvate transported into mitochondria through mitochondrial pyruvate carrier 1 (MPC1) and converted to acetyl-CoA by pyruvate dehydrogenase (PDH), a step negatively regulated by pyruvate dehydrogenase kinase 4 (PDK4). Alternatively, pyruvate can be carboxylated to oxaloacetate via pyruvate carboxylase (PC). Fatty acids undergo β-oxidation to generate acetyl-CoA, with acetyl-CoA carboxylase (ACC) regulating fatty acid synthesis. Glutaminolysis provides anaplerotic TCA substrates: glutamine is imported via SLC1A5/SLC38A1 and converted sequentially by glutaminase (GLS2) and glutamate dehydrogenase (GLUD1) or transaminases (e.g., GOT1) to α-ketoglutarate (α-KG). Within the TCA cycle, citrate synthase (CS), isocitrate dehydrogenase 2 (IDH2), and the α-ketoglutarate dehydrogenase complex (α-KG-DH) catalyze key steps, generating NADH and FADH_2_ that feed the electron transport chain (ETC). IDH2 is particularly significant as it produces mitochondrial NADPH, essential for glutathione regeneration and GPX4-mediated ferroptosis suppression. The cycle also provides intermediates for lipid metabolism: citrate exported via SLC25A1 supports fatty acid synthesis, while ACSL4 and LPCAT3 incorporate polyunsaturated fatty acids (PUFAs) into membrane phospholipids—the substrates for peroxidation. Monounsaturated fatty acids (MUFAs) counteract this process. Argininosuccinate synthase 1 (ASS1), a urea cycle enzyme, promotes reductive carboxylation of glutamine, diverting flux from oxidative TCA and reducing ferroptosis susceptibility. The ultimate balance between oxidative TCA flux (promoting PUFA synthesis and lipid peroxidation) and alternative metabolic routes (reductive carboxylation, MUFA synthesis) determines mitochondrial contributions to ferroptosis execution.

Mitochondria are both a major source of intracellular ROS and a hub for antioxidant defense. Upon ferroptosis induction, mitochondrial lipid ROS levels surge, initiating cell death [85]. Mitochondria-targeted ROS scavengers effectively reverse ferroptosis [54].

Mitochondria possess multi-layered antioxidant systems. In addition to cytosolic GPX4, mitochondria express GPX4^mito^, which directly reduces lipid hydroperoxides [54]. Two inner membrane enzymes—DHODH and GPD2—couple metabolic pathways to antioxidant defense. DHODH reduces CoQ to CoQH_2_ while catalyzing pyrimidine synthesis, generating a radical-trapping antioxidant [54,89]. Combined inhibition of DHODH and GPX4 produces synergistic lethal effects [54]. GPD2 similarly generates CoQH_2_ while oxidizing glycerol-3-phosphate [89]. MGST1, localized to mitochondria and ER, inhibits ferroptosis in an Nrf2-dependent manner [90]. AK2-mediated phosphorylation of LOXL3 stabilizes DHODH, reinforcing antioxidant defense [91].

GSH is not synthesized in mitochondria but is imported via carriers such as SLC25A10 and SLC25A11; SLC25A22-dependent NADPH synthesis supports GSH production [92]. Low mitochondrial GSH increases ROS production and GSH consumption. N-acetylcysteine (NAC), CoQ, lipoic acid, thioredoxin, and vitamins E and K exert protective effects against ferroptosis [93]. Nrf2 regulates many of these systems via antioxidant response elements (AREs) [93].

Mitochondrial fatty acid metabolism also influences ferroptosis. PC-PUFA2 interacts with the ETC to drive ROS production and lipid peroxidation [94]. Carnitine palmitoyltransferase 1 (CPT1) inhibition enhances ferroptosis, while ACC phosphorylation protects against it [95]. DECR1 knockdown induces phospholipid hydroperoxide accumulation and ferroptosis [96,97]. Lipid droplets sequester free fatty acids, protecting against PUFA oxidation [97].

## 3. Mental Disorders

Iron homeostasis is a major determinant of central nervous system function, supporting myelination, monoamine synthesis and mitochondrial metabolism. Disruption of this tightly regulated system—whether through deficiency, misdistribution or overload—has emerged as a common feature of major neuropsychiatric disorders [98,99,100,101]. Accumulating evidence now indicates that iron overload engages ferroptosis, an iron-dependent, lipid peroxidation–driven cell death pathway, thereby linking metabolic dysregulation to neuronal dysfunction and ultimately behavioral abnormalities. This emerging perspective is underpinned by a convergent molecular framework in which iron-mediated oxidative reactions, mitochondrial susceptibility, and neurotransmitter metabolism form an interconnected pathological axis.

A central component of this framework is iron’s capacity to catalyze Fenton chemistry, generating reactive oxygen species that exceed intrinsic antioxidant defenses [35]. Elevated ROS directly oxidize polyunsaturated phospholipids, triggering the lipid peroxidation processes that define ferroptotic vulnerability. Mitochondria serve both as targets and amplifiers within this cascade: ROS-mediated damage to mitochondrial membranes and mitochondrial DNA disrupts respiratory chain function, reduces ATP availability, and promotes further ROS leakage [35]. This self-reinforcing cycle not only exacerbates lipid peroxidation but also compromises ATP-dependent biosynthetic pathways, including monoamine neurotransmitter synthesis, while iron-driven oxidative modification of dopamine produces neurotoxic quinones capable of impairing mitochondrial enzymes and directly suppressing GPX4 activity [102,103]. Together, these observations support a ‘triple-hit’ model in which iron dyshomeostasis provides the initiating trigger, disease-specific metabolic or signalling disturbances supply the context, and failure of GPX4-dependent antioxidant defence unleashes lethal lipid peroxidation [104].

Despite the growing recognition of ferroptosis as a contributor to neuronal dysfunction, the specific pathways through which iron-driven cell death interfaces with distinct psychiatric conditions remain incompletely defined. The following sections examine how disease-specific etiological contexts—such as the distinct neurotoxic mechanisms of addiction, dopaminergic dysregulation in schizophrenia, or inflammatory–glutamatergic disturbances in depression—converge on ferroptotic mechanisms, offering a framework for understanding the heterogeneity of iron-associated pathology across neuropsychiatric disorders. Elucidating these relationships will be essential for determining whether therapeutic targeting of iron handling or lipid peroxidation can modify disease trajectories in clinical settings.

### 3.1. Addiction

Addictive substances converge on mesocorticolimbic dopamine signalling, but they engage ferroptosis through mechanistically distinct routes. This heterogeneity is important for understanding both selective neuronal vulnerability and the diversity of addiction-related phenotypes. Methamphetamine (METH), a prototypical psychostimulant, reverses dopamine transporter function, increases non-vesicular dopamine release, and thereby promotes cytoplasmic dopamine accumulation and auto-oxidation. The resulting ROS drives lipid peroxidation cascades via the Fenton reaction. Concurrently, METH upregulates mitochondrial iron importer Mitoferrin1 (Mfrn1) expression, causing mitochondrial iron overload, and promotes iron influx through the NMDAR-RASD1-DMT1 signaling axis, establishing a positive feedback loop of dopamine metabolism dysregulation and iron homeostasis imbalance [105,106,107]. Iron distribution in the brain is not uniform, with dopamine-rich basal ganglia being particularly susceptible to iron deposition [108]; long-term METH exposure has been demonstrated to increase iron levels in the basal ganglia [109]. Similar to cocaine, chronic METH use increases blood–brain barrier (BBB) permeability, allowing more iron to enter the brain, elevating ROS and pro-inflammatory cytokine levels in BBB endothelial cells, reducing GSH levels, and increasing lipid peroxidation—thereby inducing robust oxidative stress, lipid peroxidation, and neuroinflammation that ultimately culminate in cell death and neuronal damage [106,110]. Notably, METH-induced neuronal death exhibits parallel activation of multiple programmed cell death pathways including apoptosis, autophagy, necroptosis, pyroptosis, and ferroptosis—a characteristic that corresponds to its highest addiction transition risk (approximately 50.4%, median time 14 years) [105]. The association between cocaine and ferroptosis has also garnered significant attention. Studies have revealed disrupted brain iron homeostasis in cocaine users [111], with basal ganglia iron deposition indices positively correlated with years of drug use [111]. Brain iron homeostasis is primarily maintained by the coordinated regulation of iron transporters (transferrin receptor 1 [TfR1], divalent metal transporter 1 [DMT1], ferroportin 1 [FPN1]) and storage proteins (ferritin) [35]. Cocaine abuse disrupts this finely tuned network through multiple mechanisms: it induces oxidative stress and neuroinflammation, which upregulate TfR1 and DMT1 expression in neurons and glial cells, thereby enhancing iron uptake [106,112]; concurrently, pro-inflammatory cytokines (e.g., IL-6, TNF-α) activate the hepcidin pathway, promoting FPN1 degradation and inhibiting iron efflux, resulting in expansion of the labile iron pool (LIP) [113]. Although chronic cocaine use also increases blood–brain barrier (BBB) permeability, facilitating passive iron influx from the periphery [114], this effect is secondary to the dysregulation of iron transporters rather than the primary driver of iron accumulation. Furthermore, cocaine stimulates pro-inflammatory cytokine release and enhances oxidative stress and inflammatory responses in neural tissue, thereby accelerating iron-induced neuronal damage. Clinical studies demonstrate that individuals with cocaine use disorder exhibit reduced resistance to oxidative stress compared to non-users [106]. In contrast, opioids (morphine, heroin) induce ferroptosis through more “endogenous” mechanisms: μ-opioid receptor activation rapidly mobilizes endolysosomal iron pools for cytosolic release via Gαi signaling, subsequently upregulating ferritin heavy chain (FHC) expression. FHC not only functions as an iron storage protein but also inhibits the CXCL12/CXCR4 neurotrophic signaling axis, creating a dual “iron-dependent signaling suppression” mechanism [115]. Heroin exposure further exacerbates iron overload through PKCδ-SP1 signaling axis-mediated upregulation of transferrin receptor (TFRC) [116]. The association between alcohol and ferroptosis remains exploratory: alcohol metabolite acetaldehyde can form complexes with iron, catalyzing ROS generation, and chronic alcohol consumption affects systemic iron metabolism [106]; however, direct evidence for central nervous system ferroptosis remains lacking. Distinct addictive drugs exhibit significant differences in ferroptosis molecular mechanisms and neural circuit-selective damage, providing theoretical foundations for developing precision intervention strategies. METH preferentially affects the striatum, prefrontal cortex, and hippocampus—consistent with cognitive phenotypes including attention deficits and executive dysfunction. Morphine’s modulation of cortical neuronal dendritic spines correlates with impaired decision-making and enhanced negative affect. Cocaine iron deposition primarily localizes to the basal ganglia [105,106]. These differences suggest that ferroptosis intervention strategies require “precision matching” based on drug class and affected circuits. However, despite mechanistic studies revealing ferroptosis’s critical role in addiction neurotoxicity, causal relationships require further validation: future studies should combine genetic or pharmacological approaches to specifically intervene in ferroptosis pathways in addiction animal models, observing direct effects on drug-seeking behavior and relapse. Longitudinal clinical cohorts should track iron metabolism indicators (serum iron, ferritin) and ferroptosis markers (GPX4, SLC7A11, ACSL4) at different addiction stages, establishing correlations with cognitive function and brain structural damage [105,106]. Additionally, age and sex as potential confounding variables require consideration—brain iron levels exhibit region-specific accumulation with aging [108], and neuronal ferroptosis susceptibility may differ between developmental and aging stages; sex hormone modulation of iron metabolism may also lead to differential drug responses between males and females [107,111]. Current studies predominantly utilize young male animal models; future research should systematically incorporate age- and sex-stratified designs to more accurately assess ferroptosis’s role across different addiction subgroups. In clinical practice, individuals with addiction frequently present with psychiatric comorbidities (depression, anxiety, schizophrenia). Current single-disease studies may underestimate ferroptosis’s complex role in comorbid conditions. Bioinformatics analyses reveal that major depressive disorder-associated ferroptosis core genes (MAPK14, WIPI1, DUSP1, ULK1) correlate with immune infiltration and MAPK pathways [117]; schizophrenia patients show ferroptosis hub genes (DECR1, GJA1, SLC7A11) in prefrontal cortex involved in mitochondrial metabolism and oxidative stress [118]—pathways similarly activated in addiction-induced neurotoxicity, suggesting that ferroptosis may be synergistically amplified through shared inflammation-oxidative stress axes in comorbid states. Advancing ferroptosis mechanisms toward clinical translation requires constructing “iron metabolism indicators—ferroptosis molecules—disease severity” correlation models. Candidate biomarkers include: iron metabolism indicators (serum iron, ferritin, transferrin saturation), ferroptosis core molecules (GPX4 activity, SLC7A11, ACSL4, FTH1), and lipid peroxidation products (MDA, 4-HNE) [105,106,116]. Through weighted gene co-expression network analysis and machine learning algorithms, diagnostic models for addiction severity (craving scores, withdrawal symptoms, cognitive function) can be established and validated in independent cohorts [118]. Such models hold promise for early addiction risk warning, objective severity assessment, and dynamic treatment efficacy monitoring—ultimately facilitating integration of ferroptosis diagnostics into clinical psychiatric evaluation systems.

### 3.2. Depression

Depression has increasingly been linked to neuroinflammation, oxidative stress and impaired redox buffering, all of which may lower the threshold for ferroptosis [119,120]. Oxidative stress involves various pathological mechanisms, particularly neuroinflammation and mitochondrial dysfunction, both of which are intimately linked to the pathogenesis of depression [121,122]. Inflammation acts as an aggravating factor in the development of depression, while depression, in turn, exacerbates inflammation, creating a vicious cycle of mutual activation [119,123]. Inflammation and depression may therefore reinforce one another through partially shared pathogenic pathways, including oxidative stress and disrupted iron handling [124]. Oxidative stress arises from the excessive production of ROS and the inability of the antioxidant defence system to effectively scavenge these ROS. ROS play a pivotal role in maintaining cellular redox balance. Research indicates that the accumulation of intracellular ROS can interfere with the normal function of the 5-hydroxytryptamine (5-HT) system in mice, ultimately leading to depressive-like behaviours through the modulation of tryptophan hydroxylase-2 [125]. Therefore, ROS accumulation associated with iron metabolism abnormalities plays a significant role in the pathogenesis of depression.

Iron in the brain is primarily transported into the brain via serum iron through brain microvessels and is subsequently absorbed and utilised by neurons and neuroglial cells [126]. Epidemiological studies and animal experiments have demonstrated that various metal ions can trigger emotional dysregulation and insomnia [127]. The iron content in neurons and neuroglial cells must be maintained at an appropriate level, as excessive iron accumulation can lead to an increase in LIP and ROS, thereby causing neuronal damage.

Depression is a complex disease that is jointly determined by genetic and environmental factors. Recent studies have discovered the presence of iron oxidation in the hippocampus of mouse models of major depressive disorder (MDD), suggesting a close association between the incidence of depression and iron oxidation-related pathways [128]. Free Fe^2+^ plays a crucial role in catalyzing the formation of cellular oxygen radicals and initiating lipid peroxidation cascades by extracting hydrogen from PUFAs. Accordingly, dysregulated iron handling may contribute to both the onset and progression of depression by amplifying ROS production and lipid peroxidation. Iron ions exert a profound impact on the synthesis of neurotransmitters, the conduction of neural impulses, and the regulation of receptor functions, thereby exerting a significant influence on memory, behaviour, and cognitive functions [129].

Previous research has indicated that a reduction in GSH levels and GPX4 expression may contribute to the elevation of malondialdehyde (MDA) and ROS levels in depressed mice, which in turn may result in neuronal dysfunction and loss [119,130]. Mice subjected to chronic restraint stress exhibit symptoms of iron accumulation and iron imbalance. Furthermore, postmortem examinations of patients with recurrent major depressive disorder reveal significant decreases in GSH levels and GPX4 expression in the prefrontal cortex, providing evidence for the presence of iron oxidation in depressed patients and suggesting that modulation of iron oxidation may be a potential therapeutic strategy for depression [130]. Clinical studies further suggest that elevated lipid peroxide levels are associated with treatment-resistant depression. Consistent with this, several antidepressant interventions alleviate depressive-like phenotypes in mice while restoring hippocampal SIRT1, NRF2, HO-1 and GPX4 signalling. These studies have identified a negative correlation between depressive symptoms and high-density lipoprotein cholesterol levels. Excessive lipid accumulation can lead to an increase in ROS levels, although ROS is crucial for neuronal growth under physiological conditions [17].

Previous studies have demonstrated that depression is accompanied by decreased levels of antioxidants and increased ROS production. Antioxidants protect neurons from damage by inhibiting oxidative stress pathways and scavenging ROS. However, the specific regulatory mechanisms of ROS in depression remain incompletely understood.

Reports indicate that hydroxybutyric acid can alleviate chronic stress-induced depressive-like behaviours by inhibiting ROS levels and GPX expression. NRF2, an essential antioxidant, exhibits low expression levels in the cerebral cortex and hippocampus and serves as an important downstream target of Sirt1, which is crucial for improving the resistance of the system Xc^−^ [131,132]. Prior research has demonstrated that mice with a knockout of the NRF2 gene exhibit depressive-like behaviours, and models of chronic unpredictable mild stress demonstrate reduced NRF2 expression in the hippocampus of rats. There is an increasing body of evidence suggesting a close association between the NRF2/HO-1 pathway and recurrent major depressive disorder, indicating its significant potential in the treatment of depression [133]. Furthermore, recent studies have found that the therapeutic effects of several antidepressant drugs are closely related to the activation of NRF2. Another study has demonstrated significant alterations in the expression of GPX4, FTH1, ACSL4, and total iron in the hippocampus of mice exhibiting depressive-like behaviours [128]. Recent bioinformatic analyses have identified ferroptosis-related genes as diagnostic markers for major depressive disorder. Chen et al. constructed a diagnostic model based on three ferroptosis-related genes—ALOX15B, RPLP0, and HP—with high predictive accuracy for MDD (AUC = 0.92) [134]. ALOX15B encodes a lipoxygenase that directly catalyzes PUFA peroxidation, while RPLP0 has been implicated in both MDD and bipolar disorder, suggesting shared ferroptotic mechanisms across mood disorders [1]. Additionally, polymorphisms in GPX4 have been associated with antidepressant treatment response, with patients carrying risk alleles showing lower GPX4 expression and higher lipid peroxidation markers [119]. Together, these findings suggest that ferroptosis in depression is unlikely to reflect iron overload alone; rather, it emerges from the convergence of inflammatory signalling, redox failure and impaired neuronal lipid homeostasis.

### 3.3. Schizophrenia

In an investigation, socially isolated male rats exhibited significant disparities in iron levels within the brain when compared to rats that were housed in groups. Specifically, the former exhibited heightened iron levels in the prefrontal cortex and diminished iron levels in the hippocampus. These region-specific iron abnormalities have been associated with schizophrenia-relevant behavioural phenotypes, including anxiety-like behaviour, locomotor abnormalities and cognitive dysfunction [135].

In schizophrenia, ferroptosis has been implicated through convergent evidence linking iron misdistribution, oxidative stress, glutamatergic dysfunction and p53-related transcriptional control [136]. Further analysis of polymorphisms in this gene revealed a notable link between TP53 and schizophrenia. Researchers have proposed that the pathogenesis of schizophrenia due to this gene may be related to neurodevelopment and apoptosis [137], and the results of enrichment analyses also suggest that p53 may be involved in the apoptotic process. As a central regulator of cell death, p53 precisely regulates developmental neuronal clearance (approximately 50% of neurons are programmed to be cleared during development) in key brain regions such as the cortex and thalamus by activating pro-apoptotic genes such as BAX/PUMA [137]. The cortical thinning and thalamic volume reduction exhibited by individuals diagnosed with schizophrenia may be attributable to excessive neuronal apoptosis resulting from aberrant p53 activity. This excessive pruning has the potential to disrupt the intricate architecture of the thalamo-cortical-hippocampal neural circuit. Adaptive shifts in the pattern of p53 death regulation occur following the completion of neurodevelopmental maturation and are more frequently observed in individuals diagnosed with schizophrenia. Iron ion accumulation (Fe^2+^) in the cerebrospinal fluid of patients with schizophrenia has been shown to amplify oxidative damage via the Fenton reaction, exhibiting a significant correlation with progressive cortical/hippocampal grey matter loss [138]. By repressing SLC7A11, the core subunit of system Xc^−^, p53 reduces cystine uptake, depletes glutathione and weakens GPX4-dependent lipid repair. In parallel, activation of the p53–SAT1–ALOX15 axis promotes lipid peroxidation, thereby linking p53 signalling to ferroptotic vulnerability in schizophrenia. Within the hyperdopaminergic and NMDA-receptor-dysregulated milieu of schizophrenia, these pathways may converge to promote both ferroptotic and apoptotic damage. Genome-wide association studies further support this link. Feng et al. identified 11 differentially expressed ferroptosis-related genes in schizophrenia patients, including SLC7A11, GPX4, ACSL4, and SAT1, with SLC7A11 showing the strongest association with disease severity [139]. Lv and Luo further validated these findings, demonstrating that a ferroptosis-related gene signature could distinguish schizophrenia patients from healthy controls with high accuracy [135]. More recently, Dai et al. performed correlation analysis in three brain regions (anterior cingulate cortex, nucleus accumbens, and dorsolateral prefrontal cortex) of schizophrenia patients and identified TIMP1 and LGALS3 as potential biomarkers, with the glycolysis pathway implicated in ferroptosis regulation [140].

### 3.4. AD

**Figure 6:**
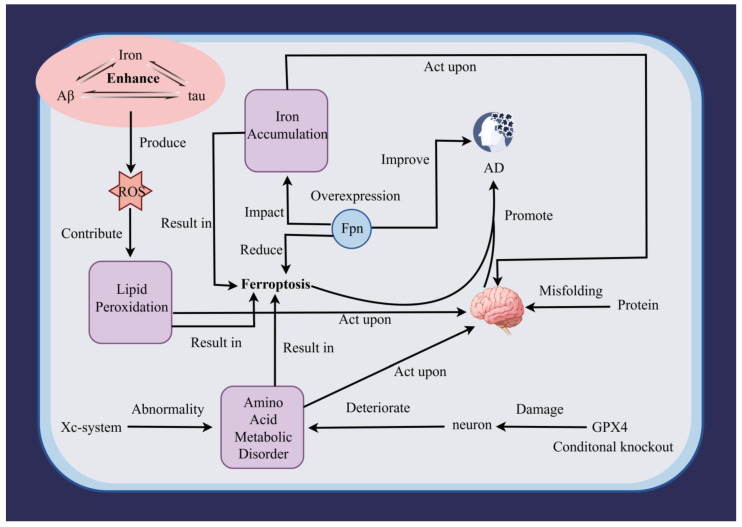
Ferroptosis and AD.

Alzheimer’s disease (AD) is neuropathologically defined by amyloid-β (Aβ) plaques and intracellular neurofibrillary tangles composed of hyperphosphorylated Tau [141,142]. Increasing evidence places ferroptosis at the intersection of iron dyshomeostasis, protein aggregation and neuroinflammation in AD [35]. Iron accumulation is a consistent finding in AD brains, with elevated iron levels detected in the hippocampus, cortex, and basal ganglia [35]. Iron dyshomeostasis in AD is reflected by altered expression of iron-storage proteins and increased TfR1-mediated iron uptake. At the same time, reduced expression or function of the iron exporter ferroportin 1 (Fpn1) limits iron efflux and expands the intracellular labile iron pool [143]; notably, Fpn1 expression levels are significantly downregulated in brain tissues from AD patients as well as in widely used AD transgenic mouse models [144]. Accumulated Fe^2+^ catalyzes the Fenton reaction, generating highly reactive hydroxyl radicals (•OH) that dramatically exacerbate oxidative stress [36], while also activating long-chain acyl-CoA synthetase 4 (ACSL4), which promotes the esterification of PUFA-containing phospholipids into cellular membranes, providing critical substrates for lipid peroxidation [16]. Aβ pathology and ferroptosis are intimately linked: Aβ oligomers bind iron to form catalytically active complexes that promote Fenton chemistry and facilitate Aβ aggregation, while Aβ deposition induces lipid peroxidation and GPX4 inactivation [145]; studies in animal models have shown that Aβ deposition can induce oxidative stress and lipid peroxidation [146], and elevated levels of the toxic aldehyde 4-hydroxynonenal (4-HNE)—a marker of lipid peroxidation—have been detected in Aβ-rich regions of AD brains, where it modifies proteins including Tau and Aβ peptides, promoting their misfolding and aggregation [143,147]. Iron directly promotes Tau pathology, as elevated Fe^3+^ levels promote liquid–liquid phase separation (LLPS) of Tau protein [148], an early step facilitating Tau condensate formation and ultimately fibrillar tangles [149]; by accelerating LLPS, iron ions lower the threshold for pathological Tau aggregation, while axonal transport deficits caused by Tau pathology impair the distribution and function of antioxidant systems within neurons, systemically weakening neuronal resistance to ferroptosis [150,151]. Neuroinflammation and ferroptosis form a positive feedback loop in AD, as pathological factors such as Aβ aggregates, pathological Tau species, and damage-associated molecular patterns (DAMPs) persistently activate microglia and astrocytes, triggering chronic neuroinflammation [152]; in activated glial cells, GPX4 expression is suppressed, rendering them susceptible to ferroptosis and causing release of pro-inflammatory cytokines (e.g., IL-1β, TNF-α), while oxidized lipid mediators generated during ferroptosis directly activate pattern recognition receptors like the NLRP3 inflammasome, amplifying the inflammatory response [153] (Figure 6).

This figure illustrates the complex bidirectional relationships linking ferroptosis to the core pathological hallmarks of Alzheimer’s disease (AD)—Aβ pathology and Tau pathology—and their collective impact on neuronal damage. Central to this network is iron accumulation, which acts as a pathogenic hub: elevated iron directly promotes Aβ aggregation and Tau misfolding while simultaneously driving ferroptosis through lipid peroxidation. Iron homeostasis is disrupted by reduced ferroportin (Fpn) expression, impairing cellular iron efflux and exacerbating intracellular iron overload. Excess iron generates reactive oxygen species (ROS) via Fenton chemistry, which in turn promotes protein misfolding and lipid peroxidation. The system Xc^−^ and glutathione peroxidase 4 (GPX4) represent key defense nodes against ferroptosis; their dysfunction—whether through direct inhibition, conditional knockout, or oxidative damage—compromises neuronal antioxidant capacity. Amino acid metabolic disorders, particularly those affecting glutathione synthesis, further sensitize cells to ferroptotic death. Aβ and Tau pathologies contribute to this cascade through multiple mechanisms: Aβ deposition enhances ROS production and lipid peroxidation, while Tau abnormalities (hyperphosphorylation, misfolding) exacerbate neuronal dysfunction. These pathological factors collectively deteriorate neuronal integrity and function. The diagram emphasizes that ferroptosis is not merely a downstream consequence but actively participates in a self-reinforcing cycle—ferroptotic cell death releases damage-associated signals that may promote further protein aggregation and neuroinflammation, while iron-driven oxidative stress simultaneously accelerates both Aβ/Tau pathology and lipid peroxidation. This integrated pathological network highlights ferroptosis as a critical nexus linking iron dyshomeostasis, protein misfolding, and neurodegeneration in AD progression.

Beyond extracellular Aβ and Tau pathology, APP processing may also shape ferroptosis more directly at the transcriptional level. The APP intracellular domain (AICD)—liberated by γ-secretase cleavage of APP—functions as a nuclear-competent transcriptional regulator of genes involved in cell death, lipid metabolism, and iron homeostasis [141]; its phosphorylation at threonine 668 (T668) is critical for interaction with Fe65 and nuclear import [154], and AICD influences ferroptosis susceptibility through regulation of FOXO3a (a master oxidative stress regulator) [155], suppression of SPTLC2 (involved in sphingolipid synthesis) [156], and interaction with appoptosin to drive heme synthesis and ROS production [157]. Association with neuropsychiatric symptoms is a key focus: depressive symptoms and anxiety in AD patients have been linked to hippocampal iron accumulation and lipid peroxidation [145]; in AD mouse models, increased hippocampal iron correlates with both cognitive deficits and depressive-like behaviors, ameliorated by iron chelation or ferroptosis inhibition [144]; clinical studies have reported that elevated serum ferritin and lipid peroxidation markers predict greater severity of depressive symptoms and faster cognitive decline [147]. Genetic evidence further supports ferroptosis involvement: the APOE ε4 allele, the strongest genetic risk factor for late-onset AD, is linked to iron dysregulation and lipid peroxidation, with APOE4 carriers exhibiting increased brain iron deposition and reduced ferritin expression [35]; polymorphisms in TFRC and SLC11A2 (DMT1) are associated with altered iron transport and AD susceptibility [143]; GPX4 variants influence AD progression, with certain alleles correlating with faster cognitive decline and elevated lipid peroxidation markers [153]; and ACSL4 expression correlates with amyloid-β burden in postmortem AD brains [16]. Collectively, these findings suggest that ferroptosis contributes to both cognitive decline and affective disturbances in AD, positioning ferroptosis inhibitors as potential dual-action therapeutics for AD-related neuropsychiatric conditions.

### 3.5. PD

In Parkinson’s disease (PD), ferroptosis provides a compelling framework for understanding the selective vulnerability of nigrostriatal dopaminergic neurons [13]. Dopamine-rich neurons in the substantia nigra are exposed to a distinctive combination of iron accumulation, oxidative stress and mitochondrial burden, creating conditions that strongly favour lipid peroxidation and GPX4 failure. Sun and colleagues showed that in the midbrain of α-synuclein A53T mice, dopamine autoxidation catalyzed by ferric iron generates dopamine quinone (DAQ), which covalently binds to the selenocysteine residue at position 102 of GPX4, triggering its NEDD4-mediated ubiquitination and proteasomal degradation [158]. This pathway is both region-specific—occurring in the substantia nigra but not in the cortex or hippocampus—and amplified by α-synuclein, providing a direct mechanistic explanation for the disproportionate vulnerability of nigral dopaminergic neurons.

The GPX4-centered antioxidant defense is further compromised by disruption of the system Xc^−^–GSH axis. SLC7A11, the core subunit of system Xc^−^, mediates cystine import essential for GSH synthesis. Zhao and colleagues showed that chronic hyperglycemia—a well-established risk factor for PD—downregulates SLC7A11 expression and function, leading to GSH depletion and sensitization to 6-hydroxydopamine-induced ferroptosis [3]. Importantly, overexpression of SLC7A11 via adeno-associated virus restored GSH levels, attenuated lipid peroxidation, and rescued both dopaminergic neuron loss and motor deficits in diabetic PD rats, highlighting a critical vulnerability node that links metabolic comorbidities to ferroptosis susceptibility [159].

Mitochondria serve as both the primary source of ROS and the primary target of lipid peroxidation in ferroptosis. In PD, complex I dysfunction—observed in sporadic cases and recapitulated in toxin models—enhances mitochondrial ROS production, which oxidizes cardiolipin and polyunsaturated fatty acid-containing phospholipids [4]. A parallel defense exists within the inner mitochondrial membrane via the DHODH-CoQ10 pathway, which reduces CoQ to the antioxidant CoQH_2_ independently of GPX4 [54,160]. CoQ10 levels are reduced in PD patients, compromising this mitochondrial ferroptosis defense; supplementation with CoQ10 or its analog idebenone has demonstrated neuroprotective effects in MPTP and rotenone models, underscoring the therapeutic potential of reinforcing mitochondrial antioxidant capacity [160].

The transcription factor nuclear factor erythroid 2-related factor 2 (Nrf2) orchestrates a broad antioxidant program that intersects with ferroptosis at multiple levels. Han and colleagues demonstrated that Acteoside, a phenylethanoid glycoside, activates Nrf2-dependent mitophagy via the PINK1/Parkin pathway in both MPTP-treated mice and MPP^+^-exposed SH-SY5Y cell [161]. This Nrf2-mediated removal of damaged mitochondria reduced mitochondrial ROS accumulation and preserved GPX4 expression, thereby suppressing ferroptosis. Conversely, pharmacological inhibition of Nrf2 or autophagy abolished these protective effects, establishing a direct mechanistic link between mitochondrial quality control and ferroptosis susceptibility [161].

Genetic determinants further modulate ferroptosis vulnerability in PD. Mutations in PLA2G6, which encodes calcium-independent phospholipase A_2_β (iPLA_2_β), cause a form of early-onset Parkinsonism with brain iron accumulation (PLAN). Li and colleagues revealed that iPLA_2_β deficiency destabilizes peroxiredoxin 6 (PRDX6), a dual-function antioxidant enzyme that interacts with ferritin heavy chain 1 (FTH1) and GPX4, leading to decreased FTH1 and GPX4 expression, iron accumulation, and heightened lipid peroxidation [162]. Treatment with the ferroptosis inhibitor Liproxstatin-1 (Lip-1) ameliorated motor deficits and dopaminergic neuron loss in PLA2G6 knockout mice, providing proof-of-concept that ferroptosis inhibition is therapeutically tractable in genetically defined PD subtypes [162]. Beyond neurons, glial cells also contribute to ferroptosis dynamics: microglial ferroptosis, regulated by SEC24B, amplifies neurodegeneration through the release of inflammatory mediators and iron, while astrocytes may undergo ferroptosis under inflammatory conditions, further compromising the antioxidant network that supports neurons [163,164].

Collectively, these studies converge on an integrated model in which the unique metabolic and biochemical landscape of nigral dopaminergic neurons creates a permissive environment for ferroptosis through the convergence of dopamine-iron synergy, glutathione dependency, mitochondrial amplification, and genetic modifiers. This framework explains why dopaminergic neurons are disproportionately vulnerable compared to other neuronal populations and identifies multiple actionable nodes—including GPX4 stabilization, SLC7A11 enhancement, Nrf2-mediated mitophagy activation, and mitochondrial antioxidant support—that hold promise for disease modification. However, several critical gaps remain: the absence of validated ferroptosis-specific biomarkers for living patients, the need for brain-penetrant and highly selective ferroptosis modulators, and the unresolved question of whether ferroptosis is an initiating event or a late-stage amplifier in sporadic PD. Addressing these challenges will be essential for translating mechanistic insights into clinically effective neuroprotective strategies for PD. PD exhibits strong genetic links to ferroptosis pathways. Mutations in PARK7 (encoding DJ-1) are associated with early-onset PD, and DJ-1 has been shown to function as a redox sensor that regulates GPX4 expression and protects against ferroptosis [143]. DJ-1 deficiency leads to increased lipid peroxidation and heightened ferroptosis susceptibility in dopaminergic neurons. Genome-wide association studies have identified SLC11A2 (DMT1) variants that modulate iron accumulation in the substantia nigra, a hallmark of PD pathology [35]. Additionally, polymorphisms in GPX4 and ACSL4 have been associated with PD risk and disease progression, with certain alleles correlating with higher lipid peroxidation markers and faster motor decline [102]. The LRRK2 G2019S mutation, the most common genetic cause of PD, has also been linked to altered iron metabolism and increased ferroptosis sensitivity [164].

Table 1 summarizes shared ferroptosis hallmarks and disease-specific mechanisms across substance use disorders, major depressive disorder, schizophrenia, AD and PD. Although all five conditions involve iron dyshomeostasis, GPX4 dysfunction and lipid peroxidation, the initiating context and dominant vulnerability nodes differ across disorders, with implications for precision therapeutic targeting.The symbols ↓ and ↑ represent the positive promotion and negative inhibition, respectively, that the relevant substances or phenomena undergo.

## 4. Strength of Evidence and Current Limitations

The strength of evidence linking ferroptosis to neuropsychiatric disease varies substantially across disorders, model systems, and levels of biological inference. Direct mechanistic support is strongest in settings where ferroptosis-specific biochemical events have been demonstrated, including GPX4 modification, suppression of system Xc^−^ activity, and accumulation of lipid peroxidation products in disease-relevant tissue. These findings support the view that ferroptosis is not merely a general consequence of oxidative stress but may participate more specifically in neuronal injury under defined pathological conditions [119,165,166,167].

At the same time, the current evidence base remains uneven, and several important limitations constrain interpretation [138,168]. First, iron dysregulation is region-specific rather than uniformly distributed across the brain, making it difficult to infer a global ferroptotic state from measurements obtained in a single structure. Second, most available data are derived from bulk tissue analyses or mixed cell populations, which obscure cell-type-specific vulnerability and prevent precise assignment of ferroptotic processes to neurons, astrocytes, microglia, or oligodendroglial lineage cells. Third, ferroptosis frequently overlaps with apoptosis, necroptosis, autophagy-dependent cell death, and inflammatory injury programmes, making it challenging to determine pathway dominance in complex neuropsychiatric settings [119,169,170]. Fourth, many reported associations between ferroptosis-related genes or markers and disease phenotypes remain correlative and do not yet establish whether ferroptosis is a causal driver, a downstream amplifier, or an epiphenomenon of broader metabolic injury [135]. Addressing these limitations will require approaches with higher spatial, temporal, and cellular resolution. Future studies should combine single-cell and spatial multi-omics, cell-type-specific genetic perturbation, ferroptosis-selective pharmacology, and longitudinal biomarker analysis in well-characterized clinical cohorts. Such strategies will be essential for defining when and where ferroptosis occurs, which cell populations are most vulnerable, and whether ferroptosis-directed interventions can produce clinically meaningful benefit.

## 5. Summary and Prospect

In this review, we position ferroptosis as a shared mechanistic framework linking iron dyshomeostasis to neuronal dysfunction across addiction, depression, schizophrenia, Alzheimer’s disease and Parkinson’s disease. Despite distinct etiologies, these conditions converge on three interconnected ferroptosis modules—system Xc^−^–GSH–GPX4 dysfunction, ACSL4–LPCAT3–ALOX-driven lipid peroxidation, and iron accumulation via TfR1–DMT1 upregulation and NCOA4-mediated ferritinophagy—all amplified by mitochondrial failure and neuroinflammation. Disease specificity emerges from the way these shared modules are engaged in different pathological contexts. In addition, vulnerability is linked to dopamine auto-oxidation and NMDAR–RASD1–DMT1 signalling; in depression, to inflammatory–glutamatergic stress and Nrf2 suppression; in schizophrenia, to p53-driven repression of SLC7A11; in AD, to AICD-linked transcriptional reprogramming; and in PD, to dopamine quinone-mediated GPX4 ubiquitination. Glial ferroptosis and gasotransmitter signaling (NO versus H_2_S) further modulate thresholds across disorders. Key unresolved questions include the temporal role of ferroptosis (initiator vs. amplifier), cell-type-specific contributions, and crosstalk with other cell death pathways. Translational barriers—lack of non-invasive biomarkers, limited brain-penetrant selective modulators, and undefined therapeutic windows—remain critical. Future priorities should focus on biomarker development (e.g., CSF oxidized phospholipids, advanced imaging), next-generation ferroptosis modulators (GPX4 stabilizers, SLC7A11 enhancers, Nrf2 activators, mitochondrial antioxidants), and precision-based strategies integrating genetic stratification and combinatorial targeting. Whether ferroptosis-directed strategies can achieve clinical impact will depend on three advances: robust biomarkers, precise patient stratification, and the development of selective brain-penetrant modulators. Together, these priorities will determine whether ferroptosis evolves from a mechanistic concept into a clinically actionable framework for neuropsychiatric disease.

## Figures and Tables

**Table 1:** Comparative ferroptosis signatures across neuropsychiatric and neurodegenerative disorders.

Dimension	Addiction	Depression	Schizophrenia	Alzheimer’s Disease (AD)	Parkinson’s Disease (PD)
**Core neurochemical characteristics**	Dopamine ↑ psychostimulants; μ-opioid receptor activation	Monoamine ↓ (5-HT, NE, DA)	Hyperdopaminergic; NMDA hypofunction	Cholinergic ↓; Glutamate excitotoxicity	Nigrostriatal DA neuron loss
**Role of iron**	NMDAR–RASD1–DMT1 ↑ iron; endolysosomal iron mobilization	Inflammation → iron retention → lipid peroxidation	DA → neurotoxic quinones; CSF iron ↑ → cortical/ hippocampal loss	Iron + Aβ → aggregation & Fenton reaction → oxidative stress	Dopamine autoxidation → DAQ; selective SN iron deposition
**GPX4 inactivation mechanism**	DAQ → Cys102 modification; NO–Dexras1– DMT1 iron influx	Glutamate inhibits Xc^−^ → GSH ↓; Nrf2 ↓	p53 → SLC7A11 ↓ → GSH ↓; p53–SAT1– ALOX15 ↑	AICD–appoptosin–heme → GPX4 ↓ via Fenton; Aβ–iron complexes	DAQ → Cys102 → GPX4 ubiquitination (NEDD4)
**Cellular/circuit consequences of ferroptosis**	Striatum, PFC, hippocampus neuronal damage; dendritic spine remodeling	Hippocampus & PFC neuron loss; neurogenesis ↓; synaptic plasticity ↓	Excessive synaptic pruning → cortical/thalamic volume ↓; GABAergic interneuron dysfunction	Pyramidal neurons ferroptosis; synapse loss → cognitive decline	Nigral DA neuron loss; striatal terminal degeneration
**Distinct** **therapeutic targets**	Iron chelators, GPX4 stabilizers, NMDAR antagonists	Xc^−^ activators (NAC), anti-inflammatory, Nrf2 activators	Antioxidants, DA stabilizers (atypical antipsychotics)	Iron chelators, GPX4 enhancers, γ-secretase modulators	GPX4 stabilizers, SLC7A11 enhancers, Nrf2 activators, mitochondrial antioxidants

## Data Availability

No new data were created or analyzed in this study.
